# Overcoming Resistance to Immunotherapy in Head and Neck Cancer Using Radiation: A Review

**DOI:** 10.3389/fonc.2021.592319

**Published:** 2021-07-01

**Authors:** Caressa Hui, Brittney Chau, Greg Gan, William Stokes, Sana D. Karam, Arya Amini

**Affiliations:** ^1^ Department of Radiation Oncology, Stanford University, Palo Alto, CA, United States; ^2^ School of Medicine, New York Medical College, Valhalla, NY, United States; ^3^ Department of Radiation Oncology, University of Kansas, Kansas City, KA, United States; ^4^ Department of Radiation Oncology, Emory University, Atlanta, GA, United States; ^5^ Department of Radiation Oncology, University of Colorado, Aurora, CO, United States; ^6^ Department of Radiation Oncology, City of Hope National Medical Center, Duarte, CA, United States

**Keywords:** radiation therapy, SBRT, stereotactic body radiation therapy (SBRT), immunotherapeutic, immune check inhibitors, head and neck cancer

## Abstract

Radiation therapy remains at the center of head and neck cancer treatment. With improvements in treatment delivery, radiation therapy has become an affective ablative modality for head and neck cancers. Immune checkpoint inhibitors are now also playing a more active role both in the locally advanced and metastatic setting. With improved systemic options, local noninvasive modalities including radiation therapy are playing a critical role in overcoming resistance in head and neck cancer. The aim of this review is to describe the role of radiation therapy in modulating the tumor microenvironment and how radiation dose, fractionation and treatment field can impact the immune system and potentially effect outcomes when combined with immunotherapy. The review will encompass several common scenarios where radiation is used to improve outcomes and overcome potential resistance that may develop with immunotherapy in head and neck squamous cell carcinoma (HNSCC), including upfront locally advanced disease receiving definitive radiation and recurrent disease undergoing re-irradiation. Lastly, we will review the potential toxicities of combined therapy and future directions of their role in the management of HNSCC.

## Introduction

Head and neck cancer is the sixth most common cancer worldwide, resulting in over 350,000 deaths per year (1). Many patients with head and neck cancer present with locally advanced disease, and radiation therapy is a mainstay of treatment for these patients, often supplemented with additional therapies including chemotherapy, surgical resection, or immunotherapy for more advanced disease (2). Radiation therapy is preferred for localized disease and is used for curative intent for a large proportion of non-oral cavity head and neck cases. Conventional radiation treatment in 2-2.25 Gy per fraction to a total of 70 Gy is the current standard for the majority of cases (3–7). In addition, retrospective data has shown that radiotherapy treatment of the primary tumor in patients with tumor recurrence and limited metastases may prolong disease-free survival (8).

Stereotactic body radiation therapy (SBRT) or stereotactic ablative radiation therapy (SABR) is another radiation modality used in select situations in the treatment of head and neck cancer. It is particularly useful for patients with recurrent head and neck tumors who have had previous radiation therapy. This is due to the smaller and more precise field of radiation applied in SBRT, leading to lower radiotoxicity and a better ability to spare normal tissue. In addition, SBRT delivers high, ablative doses of radiation therapy typically in 5 or less fractions (9). SBRT has been shown to be useful in prolonging survival while maintaining quality of life in elderly patients with unresectable head and neck tumors (10–12). SBRT has also been shown to be safe with minimal toxicity for head and neck cancer patients who are poor candidates for larger field, conventionally fractionated reirradiation (13). Overall, SBRT is a useful and safe treatment option for patients with primary, metastatic, and recurrent head and neck cancers squamous cell cancers (HNSCC) (10).

The immunological effects of radiation were first described initially in the 1970s and now there have been a vast amount of published literature describing how radiotherapy modulates the immune system. Recently, due to the advent of immune checkpoint inhibitors that have shown promising success in treating certain types of cancers, there has been mounting interest towards how radiation therapy and immunotherapy drugs can be used for a synergistic effect in treating patients. Especially in the treatment of HNSCC, the combination of immunotherapy and radiation therapy is a novel approach, therefore, there is a lack of data describing patient outcomes and toxicities.

The aim of this review is first to describe the role of radiation therapy in modulating the tumor microenvironment. Then, we will discuss how radiation therapy dose, fractionation, and target can affect the immune system and how that translates to treatment outcomes. Next, we will discuss the published data as well as ongoing studies that combine radiation therapy and immunotherapy in the treatment of HNSCC in different contexts such as: upfront definitive, re-irradiation, oligometastatic and oligoprogression. Lastly, we will review the potential toxicities of combined therapy and future directions of their role in the management of HNSCC.

## Methods

An extensive analysis of the current medical literature from peer-reviewed journals was conducted from January 1, 2008 to March 1, 2020 using the Preferred Reporting Items for Systematic Reviews and Meta-analyses (PRISMA) guidelines to systematically search the PubMed (Medline) database to retrieve a comprehensive set of relevant articles. The search strategy was developed based on National Library of Medicine^®^ Medical Subject Headings (MeSH^®^) with addition of subject-specific keywords. The bibliographies of full articles were reviewed to include studies which were potentially relevant. The literature was reviewed for quality of study design, cohort size, selection bias, evaluation of participants in relation to time from exposure, and methods of assessments. A well-established methodology (modified Delphi) was used by the expert panel to rate the appropriate use of procedures (14).

### Immunological Effects of Radiation

#### Immune System Activation

Beyond the direct effects of radiation therapy causing tumor cell death *via* DNA damage, radiation therapy has various other effects on the immune system. These include activating immune responses that lead to indirect tumor cell death *via* upregulated expression of major histocompatibility complex (MHC) class I and increased innate immune ligand expression. MHC class I molecules present endogenous peptides to cytotoxic T lymphocytes (CTL), allowing T cells to examine the peptide and induce apoptosis if the presented peptide is foreign (2, 3, 15–24). Tumor cells evade the immune system by downregulating MHC class I, so the upregulation of MHC class I by radiotherapy can cause increased cancer cell death (15, 16). Reits et al. found that MHC class I expression increased in a dose-dependent manner in two phases due to radiotherapy. The first phase was radiotherapy-produced free radicals tagging proteins for rapid degradation. The second phase was caused by mTOR kinase activation, leading to increased protein synthesis. When tumor cells were irradiated with 25 Gy, MHC class I molecules remained saturated with peptide for more than 24 hours (15, 16).

Furthermore, radiation therapy increases presentation of FAS and tumor antigens, leading to increased immune-mediated apoptosis of tumor cells. When FAS engages with its ligand, FAS-L, the apoptotic pathway is induced and caspases are activated, leading to cell death (19, 20, 25, 26). Garnett et al. showed how 91% of tumor cell lines upregulated surface molecules, including FAS, ICAM-1, CEA, MUC-1, and MHC-1 after low-dose irradiation. In addition, they showed that 6 out of 10 cell lines that expressed FAS underwent enhanced cell lysis. Furthermore, Garnett et al. observed enhanced killing of cancer cell lines by cytotoxic T lymphocytes (CTLs), even if FAS was not expressed or nonfunctional after irradiation. The apoptosis may have been due to upregulation of other molecules, such as ICAM, or increased presentation of tumor antigens by MHC class I, as discussed previously. Additionally, even nonlethal doses of radiation therapy were enough to activate the immune system, suggesting that a combination of radiation therapy and immunotherapy may be helpful even in cancers where radiation therapy is not the standard of care (16, 19). Several studies have demonstrated these effects and more between radiation therapy and the immune system (2, 3, 18–26).

#### Immune System Suppression

Radiation can be a double-edged sword in its impact on the immune system, leading to the suppression of the immune system *via* increased expression of regulatory T cells and upregulation of programmed death-ligand 1 (PD-L1) on tumor cells (2, 3, 18, 20, 24). PD-L1 overexpression by tumor cells in turn, can result in immune evasion and is overexpressed in over 50-60% of HNSCC (27). Studies have shown that regulatory T cells are more resistant to radiation than other types of T cells and may be due toan increased regulatory T cell population following radiotherapy (3, 24, 28). Regulatory T cells are hypothesized to be recruited to a tumor microenvironment in order to maintain immune homeostasis by immunosuppressive effects (3). Preclinical mouse studies done by Oweida et al. have shown significant tumor eradication in mice treated with anti-CD25 and radiation therapy when compared to mice treated with anti-CTLA4 and radiation therapy. Radiation and anti-CD25 therapy lead to tumor eradication in 57.1% of mice and improved overall survival (OS). However, tumor eradication was only achieved in mice with low levels of regulatory T cells. Therefore, head and neck cancers highly enriched with regulatory T cells are resistant to radiotherapy, even with the use of anti-PD-L1 or anti-CTLA-4 drugs. To further prove this point, the tumor was eradicated when the regulatory T cells population was depleted in combination with radiotherapy (29, 30). The combination of radiotherapy with regulatory T cell depletion is a synergistic technique that can be used to combine two antitumor modalities that are weaker on their own (3).

In addition to the suppression of the immune system *via* regulatory T cells, fractionated radiotherapy causes increased tumor cell expression of PD-L1 through production of IFN-γ (31). Normally, PD-L1 is constitutively expressed in healthy cells to prevent unnecessary killing *via* CTLs but is exploited by tumor cells to evade immune-mediated killing (2). Dovedi et al. demonstrated increased PD-L1 expression in irradiated mouse models and sequentially, an increase in tumor response to radiotherapy when also treated with a monoclonal antibody targeted against PD-1 or PD-L1. Their data shows that the combination of anti-PD-1 or anti-PD-L1 antibodies with radiotherapy can reduce tumor burden and improve survival. In addition, mice treated with monoclonal antibodies against PD-L1 and radiotherapy demonstrated significant protective tumor antigen-specific memory T-cell responses. However, treatment with anti-PD-L1 monoclonal antibodies 7 days after irradiation is completely ineffective at increasing OS when compared to only radiation therapy (31). Therefore, the timing of these two treatments must be considered for optimal tumor regression. Several other studies and clinical trials have expanded on and shown the significant anti-tumor effects of radiotherapy combined with PD-L1 inhibitors or regulatory T cell depletion (32–38).

### Radiation Delivery

#### Dose and Fractionation

Conventional radiation therapy, where low dose fractionated radiation therapy is delivered, was historically used as the standard of care in treating cancer patients. However, advances in technology have introduced the use of high ablative doses of radiation in lower number of fractions. To date, some randomized controlled trials, especially in treating patients with non-small cell lung cancer (NSCLC), have found similar outcomes in patients who are treated with SBRT when compared to conventional RT while others have even detected an improvement in survival outcomes (39, 40). Not only has SBRT quickly become a widely used option due to its improvement in survival, but also due to its favorable toxicity profile (41–44). However, its role when combined with immunotherapy is less known.

A growing body of literature suggests that improvement in survival outcomes may be attributed to dose and fractionation dependent effects on the immune system. Recent studies have demonstrated a link between radiation therapy induced lymphopenia and survival outcomes in certain cancers such as NSCLC, glioblastomas and pancreatic cancer, and lymphocyte-sparing effects have been described in patients who receive SBRT when compared to conventional RT (45–48). This raises questions of whether fractionation and dose of radiation contributes to its immunological effects through sparing immune cells which can potentially not only improve control at the primary tumor site but also at distant metastatic sites.

Preclinical data suggest that the dose of radiation delivered may have an impact on its subsequent immunological effects. Lee et al. demonstrated in a B16 melanoma mouse model that 20 Gy in 1 fraction compared to 20 Gy in 4 fractions showed considerable delay in all of the mice and complete tumor regression in 35% of the mice who received 20 Gy, but complete tumor regression in 0% of the mice who received 5 Gy x 4 (49). They hypothesized that the single fractionation leads to improvement in outcomes possibly because the fractionated RT continuously kills circulating T cells over time. Also they noted that repair of damage and proliferation between low-dose fractions could account for worse outcomes. Overall, these findings suggest conventional RT may lead to inferior RT-initiated antitumor immunity when compared to higher ablative RT doses in fewer fractions, resulting in an early relapse of tumor growth or recurrence at both local and distant sites. Although a single dose of ablative radiation is not used for curative intent, there have been impressive results described for local tumor control with high-dose single-fraction radiotherapy in palliative settings, implicating alternative mechanisms beyond the direct killing of tumor cells (50).

Conversely, Schaue et al. found that B16-OVA mice treated with doses delivered in 2, 3 or 5 fractions had better tumor responses when compared to a single fraction dose (51). They hypothesized that these findings could be due to fractionated radiation enabling the development of immunity, which is a larger factor in determining outcomes when compared to sub-lethal damage repair between treatment fractions as described by Lee et al. However, their findings partially supported Lee et al. since they also found that immune tolerance was not induced by a single dose of 5 Gy or less, which suggest that higher doses of radiation are superior to the lower doses used in conventional RT (1.5-2 Gy). Although results from Schaue et al. support that tumor immunity is improved with fractionation, it still suggests that higher doses are needed to derive an immunological benefit.

Another preclinical study by Tsai et al. found opposing results when comparing gene expression after a 10 Gy dose delivered as a single fraction *vs* 2 Gy x 5 fractions in human tumor cell lines. They found that survival after multifractionated RT was about 10 times higher than with the single dose (52). Interestingly, they found that fractionating radiation leads to selective induction of INF-related genes, which have been implicated in inflammatory and possibly radiation resistance through further induction of signal transducer and activator of transcription 1 (STAT1) (53, 54). Specifically in head and neck cancer, increased STAT1 expression have been shown to lead to radioresistance. Drugs such as fludarabine inhibit cytokine-induced activation of STAT1 and have been reported to enhance radiosensitivity of tumor cells in head and neck cancer (55). Interestingly, Khodarev et al. found that in head and neck tumor cells, STAT1, which is an upstream mediator of INF-signaling, was protective from ionizing radiation-mediated death (53). Dewan et al. also found that when combined with CTLA-4 inhibition, fractionated dosing resulted in improved control of the primary tumor when compared with single-dose RT (56). These mixed pre-clinical results suggest multiple complex variables other than dose fractionation contribute to the immunological effects of radiation therapy.

Lastly, another fractionation alternative may be hypofractionated treatment typically given in the range of 15 to 20 fractions. Further work in the arena of combined immunotherapy and hypofractionated RT for head and neck cancer is needed.

#### Field Size and Elective Nodal Coverage (ENI)

Not only does SBRT utilize a higher dose per fraction, which may be associated with improved outcomes in patients with oligometastasis through various immunological effects as noted above, but also offers decreased toxicity and immunosuppression through a high degree of dose conformity when compared to conventional radiation therapy which normally encompasses larger areas. Decreased margins in SBRT when compared to conventionally fractionated radiation therapy theoretically decrease radiation to healthy tissue, which could blunt destruction of lymphocytes that are necessary to illicit an anti-tumor response.

Oftentimes, treatment fields include the primary tumor as well as clinically uninvolved draining lymph node regions that are at high risk for micrometastases to prevent local recurrence. Elective nodal irradiation is currently the standard of care for the majority of head and neck cancers based on the high rate of spread to regional lymph nodes. However some studies in other tumor types suggest that large field nodal radiation may not add significant improvement in cancer outcomes. For example, in several randomized trials and large dataset analyses, survival outcomes of whole-pelvic *vs* prostate-only radiation therapy for high risk prostate cancer were no difference (57–59). Rwigema et al. showed that in patients with early-stage NSCLC, prophylactic RT to the mediastinum did not improve outcomes (60). Not only have the studies above found a lack of improvement in survival outcomes, but also, Marciscano et al. has found that ENI actually adversely affected survival outcomes when combined with immune checkpoint blockades due to altering adaptive immune responses such as chemokine expression and CD8+ T-cell trafficking (61).

The question of the use of ENI has come into a new light, specifically in respects to its effect on the immune system, as multiple studies have described the negative effects of treating draining lymph nodes on the efficacy of immunological responses. Studies have reported that radiation therapy to the local tumor elicits different immunomodulatory effects that lead to immune mediated tumor-specific responses. Lugade et al. showed that radiation increases IFN-γ–producing antitumor immune cells and Apetoh et al. demonstrated that T cells in radiation therapy decrease tumor growth by comparing T-cell deficient mice models and wild-type mice, while Lee et al. showed that CD8+ T cells in radiation therapy resulted in tumor growth inhibition (49, 62, 63). Taken together, CD8+ T cells, IFN-γ–producing antitumor cells, and T-cell proliferation in the tumor draining lymph node (DLN), lead to tumor-specific responses. Similarly, Takeshima et al. demonstrated in their animal models that tumor DLN were required for inducing tumor-specific CTL and found that CTL were significantly decreased by radiating the DLN, which lead to significantly worse survival outcomes (64). These findings were observed in lymph node deficient mouse models, and tumor-specific CTLs are indispensable in creating tumor specific CD8+T cell responses (65, 66). Sharabi et al. showed that locally directed radiation therapy increases the activation and proliferation of an antigen-specific antitumor T-cell population in the DLN, which proposes the question that SBRT may lead to improved outcomes when compared to conventional RT by sparing destruction of T-cells in the DLN (33).

The pre-clinical findings above suggest that omitting ENI may assist immunological responses that can potentially be further enhanced by the use of immune checkpoint blockades. However, although several clinical studies mentioned above fail to demonstrate the benefits of ENI in certain cancers, the treatment of DLN specifically in head and neck cancer warrants additional investigation due to the extensive and complex lymphatic drainage in the area. Currently, the standard for upfront locally advanced head and neck cancer is to cover elective lymph nodes whereas it is not recommended in the setting of re-irradiation.

### Radiation Combined with Immunotherapy

#### Mechanisms

Treatment of HNSCC typically involves a multidisciplinary approach composed of surgery, radiation therapy, and chemotherapy. Patients with localized disease are generally managed with either radiation therapy or surgery, but patients with more advanced cancers are managed with multimodality approaches. Despite the use of multimodality treatment, outcomes and prognosis for metastatic head and neck cancer remain poor. There has been a growing number of studies using immune checkpoint inhibitors in recurrent or metastatic head and neck cancer and the results of the first clinical trials using PD-1/PD-L1 drugs have shown a survival benefit along with a favorable toxicity profile when compared to standard treatments (67–71). The improved treatment responses and clinical outcomes in these studies shed light on the importance of not only understanding the mechanism of immune checkpoint inhibitors but also of their interaction with other treatment modalities.

Radiation therapy induces apoptosis, necrosis, and senescence of tumor cells through inducing DNA damage by directly causing breaks in the DNA strands, or indirectly by reactive oxygen and nitrogen species (72). The advantage of delivering a conformal dose to the tumor while minimizing systemic toxicity and sparing neighboring healthy tissue makes radiation therapy an attractive choice for multimodality treatments. In addition to DNA damage, preclinical studies substantiate that radiation can induce tumor-specific immunity and contribute to immunogenic cell death (73). Now, several robust preclinical studies support the synergistic effects of systemic immunotherapy and radiation therapy. Reits et al. found that when radiation was combined with adoptive CTL therapy in an MC38 colon cancer model, tumor growth inhibition was significant increased when compared to either modality alone (15). Furthermore, Zhang et al. found that in mouse models, radiation therapy alone was insufficient to eradicate the cancer, but when radiation of the MC57 tumors were followed by CTL transfer, the tumor was eradicated (74).

In addition to these preclinical studies, Demaria et al. used 4T1 mouse models to determine if immune checkpoint blockade can act synergistically with radiation therapy to delay tumor growth. They found that the combination of radiation therapy and a CTLA-4 antibody improved OS and antitumor activity. Radiation therapy alone only slowed primary tumor growth and anti-CTLA-4 therapy alone did not improve survival outcomes or delay tumor growth. Furthermore the control of distant metastases observed in mice who received the combination therapy was immune-mediated and dependent on CD8+ T cells (75). Additionally, other preclinical studies have proposed mechanisms that could possibly account for the synergistic affect seen in radiation therapy and immunotherapy. Ruocco et al. found that in the 4T1 model, MCH-1 dependent arrest was restored after treatment with radiation therapy and a CTLA-4 blocking antibody, which allowed improved antitumor activity through the interaction of tumor-infiltrating lymphocytes and tumor cells (76). Additionally, Belcaid et al. found that in an orthotopic mice models of glioma treated with triple therapy including 4-1BB activation, CTLA-4 blockade, and radiation therapy these mice had increased survival, and a higher density of CD8+ and CD4+ cells. When mice had depletion of CD4+ T cells, the antitumor efficacy of triple therapy was abrogated, highlighting the importance of CD4+ T cells in the synergistic effect, and this was independent of the sequence of the treatments delivered (77).

Preclinical work has also included studies on PD-1 inhibition with radiation therapy. Verbrugge et al. observed that PD-1 is an indispensable signal in mediating the antitumor response of radiation therapy in a triple-negative breast cancer model. They found that all mice were cured when a PD-1 antibody was combined with single or low-dose fractionated radiation therapy and that CD8+ T cells were essential for this curative response. In a CT26 murine colon cancer cell line, Dovedi et al. found that 10 Gy delivered in 5 fractions concurrently delivered with anti-PD-L1 therapy improved OS (31). Unlike the findings by Belcaid et al, the improvement in OS in these CT26 models was dependent on the sequence of treatment delivered and was only seen when anti-PD-L1 therapy was given concurrently with radiation therapy (31). Mechanisms have been proposed to explain this synergistic effect between radiation therapy and anti-PD-L1 therapy as well. Deng et al. demonstrated that radiation causes an up-regulation in PD-L1 in the tumor microenvironment, so the addition of a PD-L1 blockade to radiation causes an activation of cytotoxic T cells (32). Additionally, Sharabi et al. suggests that radiation with anti-PD-1 therapy incudes an antigen-specific immune response as discussed above (20).

Thus as a result of these robust preclinical studies detailing synergistic effect between radiation therapy and immunotherapy, many ongoing clinical trials are investigating the use of radiation with immunotherapy. However, the optimal sequence of treatment delivery, the radiation dosing and fractionating, and patient selection to best illicit this synergistic effect in head and neck cancer remains unknown. Current published data is limited, but this will likely increase due to the high number of ongoing clinical studies.

#### Upfront Definitive Radiation Therapy

Published data is sparse and many trials are still actively accruing patients (**Table 1**); however, the phase III JAVELIN 100 trial, which is a randomized, double-blind, international multicenter trial, comparing avelumab plus chemoradiation versus standard of care chemoradiation in patients with locally advanced HNSCC has been terminated after a planned interim analysis. The trial aimed to demonstrate that the combination of avelumab with standard chemoradiation offers superior progression-free survival (PFS) when compared to chemoradiation alone in the treatment of patients with high-risk, locally advanced HNSCC. At the interim analysis, they concluded that the study is unlikely to show a statistically significant improvement in their primary endpoint of PFS. So far, the phase III JAVELIN Ovarian PARP 100 trial and the JAVELIN 100 trial for HNSCC has been terminated, but the JAVELIN Merkel 200 trial is still active, as it shows durable responses and meaningful survival outcomes in patients with Merkel cell carcinoma at 2 years (78).

**Table 1:** Select Ongoing Trials/Awaiting Results.

Trial	Phase	N	Eligibility	Regimen Studied	Control Arm	Primary Endpoints	Secondary Endpoints	Estimated Completion Date
**JAVELIN 100 **	III	697	- HPV-, Stage III-IVb - Non-oropharyngeal HPV+ Stage III-IVb - HPV+ oropharyngeal disease T4 or N2c or N3	Control + concurrent and adjuvant Avelumab for 12 months	70 Gy in 35 fractions + q3 weeks cisplatin 100 mg/m2	PFS	OS, pCR, LRF, ORR, DM, DOR, AE, QOL	6/2020
**JAVELIN Merkel 200**	II	204	Metastatic Merkel Cell carcinoma	First line avelumab, and second line avelumab	None	BOR, DRR	DOR, PFS, AE, OS	5/2024
**HN003 **	I	37	Stage III-IV HNSCC	RT + qweekly cisplatin + q3 weeks pembrolizumab	None	DLT	DFS, OS, LRF, DM, AE	10/2018
**HN005**	III	711	Stage T1-2, N1, or T3, N0-N1, M0 p16+ oropharyngeal cancer	60 Gy in 6 weeks + q3 weeks cisplatin 100 mg/m2 *vs* 60 Gy in 5 weeks with nivolumab	70 Gy in 6 weeks + q3 weeks cisplatin 100 mg/m2	PFS, QOL	LRF, DM, OS, AE	2/2025
**HN004**	II/III	474	Stage III-IV SCCA of the head and neck with a contraindication to cisplatin	RT + cetuximab *vs* RT + durvalumab	None	DLT, PFS, OS	LRF, DM, PET response, AE, AOL	12/2025
**EA3161**	II/III	744	Oropharyngeal cancer p16+: - Stage T1-2N2-3 or T3-4N0-3 with ≥ 10 pack years - Stage T4N0-N3 or T1-3N2-3 <10 pack years	RT + qweekly cisplatin + adjuvant nivolumab	RT + qweekly cisplatin	PFS, OS	Effect of PD-L1 expression, HPV status, SUVmax on OS and PFS, PET response	1/2027
**HN007**	III	316	Recurrent nasopharyngeal cancer	Nivolumab, gemcitabine, and cisplatin or carboplatin	Gemcitabine and cisplatin or carboplatin	OS	LRF, DM, PFS, ORR, AE, QOL	5/2028
**EA3191**	II	282	Recurrent or second primary HNSCC in a previously radiated field	RT + pembrolizumab or pembrolizumab alone	RT + cisplatin or carboplatin	OS, AE	DFS	2/2026

ORR, objective response rate; OS, overall survival; pCR, pathologic complete response; LRF, locoregional failure; DM, distant metastasis; DOR, duration of response; AE, adverse events; QOL, quality of life; BOR, best overall response; DRR, durable response rate; DFS, disease free survival; DLT, dose limiting toxicities.

The NRG recently completed the HN003 trial, which was a phase I study in patients with HPV negative, stage III-IV HNSCC where pembolizumab is administered concurrently with postoperative radiation therapy and weekly cisplatin. This study aims to compare outcomes of these patients to the current standard of care with the primary outcome being dose-limiting toxicities up to 4 weeks posttreatment (79). Secondary outcomes include change in expression of peripheral immune inflammatory biomarkers, levels of PD-L1, and survival and disease control outcomes at 1 year. This trial is currently undergoing scheduled interim analysis. Another NRG trial, HN005, is a randomized phase II/III trial that studies the outcomes of patients who are given a reduced dose of radiation therapy with nivolumab compared to standard dose of radiation therapy plus cisplatin in treating patients with HPV positive early stage oropharyngeal cancer. Patients will either be given intensity modulated radiation therapy (IMRT) over 6 fractions per week and receive cisplatin, versus reduced dose IMRT over 5 fractions per week with cisplatin, versus nivolumab plus reduced dose IMRT over 6 fractions per week. These trials aim to explore the use of up front immunotherapy in patients with HNSCC and will be important in determining the role of these checkpoint inhibitors in treating HNSCC.

A number of studies are ongoing in this area to identify novel immune checkpoint inhibitors to be delivered concurrently with radiation therapy (80). Currently the only targetable agent combined with radiation treatment in definitive head and neck cancer is cetuximab, an epidermal growth factor inhibitor (EGFR), which showed improved survival outcomes when compared to radiation alone (81). However recent randomized data for HPV positive patients suggest inferior outcomes when compared to cisplatin (82, 83).

#### Re-Irradiation

Patients with head and neck squamous cell carcinoma who recur after definitive therapy have limited treatment options; specifically those who fail platinum-based chemotherapy have a survival of less than 6 months (84). Options include: surgery, but this may be precluded by the extent of the recurrence; chemotherapy, which has a poor prognosis; and re-irradiation depending on the previously treated field and the site of recurrence. For example, for patients with metastatic HNSCC or recurrent disease that is unable to be treated with a curative intent, the current standard of treatment pembrolizumab with or without chemotherapy (85).

With the recent successes in immunotherapy for the treatment of other cancers, and promising outcomes for the use of radiation therapy and immunotherapy upfront in HNSCC, now several ongoing studies seek to elucidate the outcome of immunotherapy combined with re-irradiation in patients with recurrent disease (86). The KEYNOTE-012 trial was the first trial to show efficacy of immune checkpoint inhibitors in HNSCC. This was a multicohort phase Ib study to evaluate not only the efficacy by the safety of pembrolizumab in patients with advanced solid tumors, including patients with recurrent and metastatic HNSCC. This cohort of patients was initially divided into 60 patients with PD-L1 positive tumors (≥1% PD-L1 expression). Patients received pembrolizumab 10 mg/kg IV every 2 weeks and the objective response rate (ORR) was 18% in all patients, 25% in HPV positive patients and 4% in HPV negative patients. 17% of these patients had grade 3-4 drug-related adverse events, and 45% of patients experience a serious adverse event. There were no drug-related deaths. The duration of response was about 53 weeks and OS was 13 months (67). The expansion cohort enrolled an additional 132 patients with recurrent/metastatic HNSCC regardless of PD-L1 expression status. Pembrolizumab was given once every 3 weeks instead of 2 weeks, and the ORR in this patient population was found to be 18% in all patients, 32% in HPV positive patients and 14% in HPV negative patients. PD-L1 status was predictive of ORR (22% for PD-L1 positive *vs* 4% in PD-L1 negative patients) (68). In the pooled analysis of the initial and expanded cohort, 17% of patients achieved stable disease, median OS was 8.5 months, and the 6-month PFS rate was 24.9% (87). Overall, Keynote-012 concluded that pembrolizumab was well tolerated with good clinical outcomes, and should be strongly considered in patients with recurrent/metastatic HNSCC. Based on the results found in this trial, pembrolizumab was approved by the US Food and Drug Administration (FDA) in the treatment of recurrent and metastatic HNSCC in 2016. Currently we are awaiting the results of Keynote-040, which is a recent ongoing phase III trial that has reached accrual. 466 patients with recurrent/metastatic HNSCC were enrolled and patients were randomized to treatment with either pembrolizumab *vs* methotrexate, cetuximab or docetaxel.

Checkmate-141 is a randomized phase III trial with 361 patients with recurrent HNSCC who progressed after platinum-based chemotherapy. This study aimed to evaluate whether nivolumab improves OS when compared another therapy of the investigator’s choice (including either docetaxel, methotrexate, or cetuximab). They found that nivolumab significant improved OS when compared to other therapies (7.5 months *vs*. 5.1 months, respectively), and the 1 year OS rate was greatly improved in patients treated with nivolumab versus standard therapy (36.0% versus 16.6%, respectively). There was a 30% reduction in risk of death for patients treated with nivolumab. Interestingly, regardless of PD-L1 expression or p16 status, OS was improved in patients treated with nivolumab when compared to patients treated with standard therapy, although patients with PD-L1 positive or HPV-positive status benefited the most. Overall response rate was 17% in PD-L1 positive patients, 12.3% in PD-L1 negative patients, and 15.9% in patients with p16 positive disease versus 8.0% in patients with p16 negative disease. Drug related adverse events were significantly lower in the nivolumab group (59.3%) versus the standard care group (77.5%). This trial concluded that nivolumab had a lower incidence of drug related adverse events and improved OS when compared to the standard therapy (70).

The KEYSTROKE trial hopes to explore the synergistic effects of re-irradiation with PD-1/PD-L1 inhibitors and clinical outcomes of patients treated with SBRT plus anti-PD-1 therapy versus SBRT alone. Patients eligible for the KEYSTROKE trial must have pathologically confirmed diagnosis of locoregional recurrent or unresectable new primary HNSCC, must have had prior radiation therapy to the head and neck to a minimum of 30 Gy with overlap of at least 25% of the current planned tumor volume (PTV) with the previously treated area, and the disease must be limited to a single site or adjacent sites that can be treated in a single contiguous target volume for which the gross tumor volume (GTV) must be <7.5 cm (86). Patients will be randomized to receive pembrolizumab plus SBRT versus SBRT alone. Additionally, the REPORT trial is aiming to study the outcomes of patients with recurrent HNSCC who have had prior radiotherapy and will randomize patients with receive with nivolumab alone or nivolumab plus radiation therapy delivered to a total dose of 60 Gy in 1.5 Gy fractions BID for 4 weeks (88). Similarly, a phase II trial currently accruing patients will randomize patients to reirradiation only of 1.2 Gy BID for 5 weeks versus pembrolizumab in addition to reirradiation in patients with locoregional inoperable recurrent HNSCC or second primary HNSCC (**Table 1**) (89).

### Potential Toxicities

There is a paucity of published clinical data as noted above regarding the efficacy and survival outcomes in patients with HNSCC treated with immunotherapy and radiation therapy. There is more available data however, regarding the safety profile and potential toxicities of combining immunotherapy and radiation therapy. The GORTEC 2015-01 phase II trial accrued 133 patients with inoperable stage III-IVb HNSCC who were unable to tolerate cisplatin and randomized them to receive either cetuximab with RT or pembrolizumab and RT. Although the efficacy results are still pending, they found that the tolerance of pembrolizumab plus radiation was better when compared to cetuximab plus radiation (90). However, they found that treatment-related mortality was slightly higher in both arms when compared to previous GORTEC studies, which may be confounded by differing baseline characteristics, as these the inclusion criteria of this study included patients who cannot tolerate cisplatin, which could be a surrogate for poorer baseline function.

Wise-Draper et a. recently reported the preliminary safety data of the ongoing phase II trial (NCT02641093) which enrolled patients with locally advanced resectable HNSCC. These patients received one dose of pembrolizumab followed by surgery and all patients received either adjuvant concurrent pembrolizumab plus radiation therapy versus pembrolizumab plus cisplatin plus radiation therapy in patients with high risk features. At the interim analysis, no grade 4 toxicities or dose limiting toxicities were observed (91).

Furthermore, the safety of pembrolizumab with chemoradiation in locally advanced HNSCC was reported by Powell et al. who showed that all patients completed radiation therapy without delay, while 3 patients of 27 discontinued immunotherapy due to grade 2 peripheral neuropathy, grade 1 Lhermitte syndrome, or grade 3 elevation in liver enzymes (92). In RTOG 3504 trial, patients with intermediate risk HNSCC were treated with nivolumab in addition to chemoradiation. At the time of the interim analysis, 3 of the 17 patients discontinued cisplatin, 3 patients discontinued nivolumab for known side-effects of the drug, and only one grade 4 adverse event of elevated amylase was seen but resolved (93). These trials above support the safety of combined immunotherapy with radiation therapy. In the oligometastatic setting, combination of radiation therapy with immunotherapy does not appear to have increased rates of immune-related adverse events or significantly effect quality of life (94, 95).

So far, in HNSCC and in other disease sites, immunotherapy plus radiation therapy exhibits a favorable toxicity profile and is well tolerated. Specifically, the safety of SBRT combined with immunotherapy in metastatic HNSCC was studied in a phase II trial where patients either received nivolumab alone or nivolumab with SBRT, which was delivered as 9 Gy x 3 fractions. They found that the rates of grade 3 or greater toxicities were low in both arms (96). Overall, these studies support that immunotherapy combined with radiation therapy is well-tolerated in HNSCC patients (**Table 2**).

**Table 2:** Select Preclinical and Clinical Data Summary.

Preclinical Data	Clinical Data
When radiation is combined with CTL therapy, tumor growth inhibition was increased Tumor infiltrating lymphocytes and tumor cell interaction was increased after treatment with radiation therapy and a CTLA-4 blocking antibody CTLA-4 blockade plus radiation therapy in mice lead to a higher density of CD8+ and CD4+ cells. When CD4+ T cells are depleted, the antitumor activity decreased When PD-1 antibody was combined with radiation, mice with triple negative breast cancer were cured Radiation plus a PD-L1 blockade causes an increased activation of cytotoxic T cells	Checkmate-141: 1 year OS was improved in patients with recurrent HNSCC who progressed after first line chemotherapy that received nivolumab KEYNOTE-012: PD-L1 status is predictive of the ORR in patients with recurrent/metastatic HNSCC KEYNOTE-040: median OS is improved in patients with metastatic or recurrent HNSCC who received pembrolizumab. Patients with at least 1% PD-L1 benefited more form pembrolizumab KEYNOTE-048: Pembrolizumab alone is noninferior when compared to cetuximab. Pembrolizumab with chemotherapy improved OS compared to cetuximab with chemotherapy. Patients with PD-L1 expression benefited more from pembrolizumab

## Conclusion

Radiation therapy remains at the center of head and neck cancer treatment. With improvements in treatment delivery, radiation therapy has become an effective ablative modality for head and neck cancers. Further, radiation appears to play a large role in activating immune responses and may be the spark needed to improve the efficacy of novel immune checkpoint inhibitors coming down the pipeline. With improved systemic therapies, local noninvasive modalities such as radiation are critical in overcoming resistance in head and neck cancer. Data from ongoing trials and future studies are needed to better understand the mechanism of radiation and immune checkpoint inhibitors, how best to sequence the therapies, what dose of radiation is most optimal, and what areas should be targeted. As data further matures in head and neck cancer research, it will become even more critical that these patients are discussed and treated in a multidisciplinary fashion.

## Author Contributions 

CH, BC, and AA collected the data and wrote the majority of the manuscript. All authors contributed to the article and approved the submitted version.

## Conflict of Interest

The authors declare that the research was conducted in the absence of any commercial or financial relationships that could be construed as a potential conflict of interest.
